# Computational analysis of peripheral blood RNA sequencing data unravels disrupted immune patterns in Alzheimer's disease

**DOI:** 10.3934/Neuroscience.2024007

**Published:** 2024-04-19

**Authors:** Dimitra Anatolou, Marios G. Krokidis

**Affiliations:** 1 Bioinformatics and Neuroinformatics MSc Program, Hellenic Open University, Patras, Greece; 2 Bioinformatics and Human Electrophysiology Laboratory, Department of Informatics, Ionian University, Corfu, Greece

**Keywords:** immune response, Alzheimer's Disease, RNA-sequencing data, enrichment analysis, neuroinflammation

## Abstract

The central nervous system (CNS) and the immune system collectively coordinate cellular functionalities, sharing common developmental mechanisms. Immunity-related molecules exert an influence on brain development, challenging the conventional view of the brain as immune-privileged. Chronic inflammation emerges as a key player in the pathophysiology of Alzheimer's disease (AD), with increased stress contributing to the disease progression and potentially exacerbating existing symptoms. In this study, the most significant gene signatures from selected RNA-sequencing (RNA-seq) data from AD patients and healthy individuals were obtained and a functional analysis and biological interpretation was conducted, including network and pathway enrichment analysis. Important evidence was reported, such as enrichment in immune system responses and antigen processes, as well as positive regulation of T-cell mediated cytotoxicity and endogenous and exogenous peptide antigen, thus indicating neuroinflammation and immune response participation in disease progression. These findings suggest a disturbance in the immune infiltration of the peripheral immune environment, providing new challenges to explore key biological processes from a molecular perspective that strongly participate in AD development.

## 1. Introduction

Alzheimer's disease (AD) emerges as the predominant form of late-onset dementia, imposing profound societal and individual impacts. Characterized by a progression, the disorder involves neurodegeneration occurring in the brain decades before the diagnosis [1]. The extensive gap between the onset of initial pathophysiological changes and the manifestation of clinical symptoms implies the existence of an AD continuum, thus delineating various transitional stages. A prodromal stage known as mild cognitive impairment (MCI) precedes the dementia phase, marked by cognitive deficits that, while present, do not significantly impede daily activities. Further back in the continuum, preceding even MCI, is the preclinical AD phase [2],[3]. Consequently, there is an urgent need for early markers to discern the silent neurodegeneration that occurs before the clinical signs of dementia manifest.

The ultimate physiological reaction to chronic stress engages the immune system, thereby triggering the production of pro-inflammatory cytokines. These molecules have the capacity to directly affect neural activity in the brain [4], play a role in the inflammatory response, and have a direct impact on the neural activity in the brain. Chronic stress can lead to a dysregulated immune response, contributing to inflammation that may influence various physiological processes, including neural function. In humans, chronic stress experienced during early life or adulthood also exerts an influence on the recognition and development of various psychopathologies. However, individual differences underscore the belief that factors such as sex and genetic makeup may contribute to the development of distinct stress-related mental health disorders, including mild cognitive impairment and AD [5]. Aging triggers inflammation, which involves microglia activation and disrupted circadian rhythms due to cortisol signaling. This disruption leads to altered circadian rhythms, and sleep disturbances contribute to increased cortisol secretion [6]. Cognitive and affective neural networks may undergo aging-related changes linked to the interplay between these systems and the adrenal medulla, which is the final component of the hypothalamic-pituitary-adrenal (HPA) axis.

Chronic stress, via HPA-axis dysregulation, can trigger co-morbid depression in neurodegenerative diseases [7]. Chronic inflammation is a key factor in AD pathophysiology, thereby disrupting adult neurogenesis. Studies consistently show decreased neurogenesis rates in AD, which was linked to β-amyloid. Inflammatory components regulate neural stem cell processes, in which elevated levels contribute to an inflammatory response in AD, thus leading to the loss of neurons and synapses in the cerebral cortex [8]. Within the brain, immune cells such as microglia and lymphocytes play a substantial role in shaping cognition and neuronal circuits [9]. Despite similarities, differences exist in target recognition and cell dynamics, shaping their distinct roles and functions. The immune system's malfunction is closely linked to AD progression. Extensive evidence shows that immune cells, particularly microglia in the central nervous system (CNS) and peripheral blood-derived cells, undergo dynamic changes and varied functions at different disease stages [10]. In the present study, a differential expression analysis was performed on selected RNA-sequencing (RNA-seq) data obtained from both AD patients and healthy controls, followed by a biological interpretation using an enrichment analysis to extract important biological insights relating to gene functionality.

## 2. Materials and methods

### 2.1. Dataset curation

Our analysis was applied to the peripheral blood mononuclear cells (PBMCs) generated by Gagliardi et al. [11] (GEO accession number: GSE203408). This dataset contains RNA-seq data that was derived from AD patients and healthy controls. Moreover, this particular study includes data from samples before and after treatment with a nanoformulation; however, we only used data derived from untreated samples since our objective was to unravel the most dominant genes that differentiated AD patients from healthy individuals. In particular, transcriptomic data from 4 patients with an average age equal to 72.2 years old (25% male, 75% female) and 3 healthy controls with an average age equal to 63 years old (33% male, 67% female) were included in the analysis.

### 2.2. Differential Expression Analysis

The selected data were analyzed for differential expression using the RaNA-seq platform [12]. RaNA-seq is a cloud-based RNA-seq analysis platform that filters and quantifies FASTQ data, computes quality control measures, and performs differential expression and functional analyses, thus enabling a full analysis of data in a matter of minutes [12]. Its pipeline follows quantification and quality control of all samples with a simple error ratio estimate (SERE), which is a single-parameter test to count data and estimate the error rate in a sample by examining the ratio of errors to the total observations in that exact sample [13]. Quantification and alignment of reads per gene were performed using Salmon, which is a precise and quick method to quantify the transcript abundance from RNA-seq reads [14]. Then, raw RNA-seq read counts were normalized and the differentially expressed genes (DEGs) between the control and patient samples were extracted with the DESeq2 method of median ratios [15]. DESeq2 is a powerful bioinformatics tool used to identify DEGs between different conditions or treatments by employing negative binomial modeling to account for variability in sequencing data, normalization techniques to remove systematic biases, and statistical tests to assess the significance [15]. The Wald test and parametric fit type adjusted p-value (Benjamini-Hochberg) cutoff were set at 0.05. Statistically significant DEGs were deemed all genes with a |log2FC| ≥ 1 and an adjusted p-value ≤ 0.05.

### 2.3. Network and Pathway Enrichment Analysis

A protein-Protein Interaction (PPI) network of the DEGs was created using STRING, which is a platform that integrates information from various sources, such as experimental data, computational predictions, and existing databases, to construct comprehensive protein interaction networks [16]. All available interaction sources, a medium confidence (0.4), and the Markov Cluster algorithm (MCL) with an inflation parameter equal to 3 were selected for the construction and clustering of the network. The MCL algorithm operates by simulating random walks through a graph, thereby emphasizing strong connections between nodes and iteratively identifying clusters based on dense regions. Different open source and freely available enrichment analysis tools were used to identify pathways related to the identified DEGs, using only the statistically significant DEGs as the input: Enrichr [17] and GeneCodis [18]. Moreover, the STRING-produced network was exported to Cytoscape [19] for connectivity and pathway analyses. The Gene Ontology Biological Process (GO BP), the Molecular Function (GO MF), the Cellular Component (GO CC), and the KEGG pathway database were selected for the membership and enrichment analyses. Finally, the results provided by each tool were compared to each other.

## 3. Results and discussion

According to our process, the differential expression analysis revealed 49 DEGs, 25 up- and 24 down-regulated, as shown in Table 1. The most dysregulated genes are members of the human major histocompatibility complex (MHC) (HLA-E, -A, -C, -DRB1, -DQA1, -DQB1). Members of the MHC superfamily known to be important for antigen presentation play a crucial role in the immune system and its response to stress, thus proposing a strong connection to many stress-related disorders [20],[21]. Many studies have focused on identifying the polymorphisms carried by AD patients on these genes, though with no convincing association [22]. However, studies on the immune profile of AD patients have revealed an excessive activation of cytotoxic T cells [23], suggesting an alteration in the expression of MCH-related genes.

**Table 1.** Statistically significant dysregulated genes between AD patients and healthy controls.

**Gene**	**Ensembl ID**	**Log2FC**	**p-adj**
**Downregulated DEGs**
HLA-E	ENSG00000204592.8	-7.72	2.5e-65
HLA-A	ENSG00000223980.10	-7.325	4.8e-5
HLA-DRB1	ENSG00000228080.10	-7.007	0.014
HLA-A	ENSG00000206505.13	-6.938	1.6e-17
FLOT1	ENSG00000236271.8	-4.333	0.025
CHIT1	ENSG00000133063.15	-4.263	0.033
HLA-DQA1	ENSG00000228284.8	-4.032	0.007
TRIM27	ENSG00000233948.10	-3.723	0.006
ZPBP2	ENSG00000186075.12	-3.715	0.014
AC044810.9	ENSG00000284597.1	-3.679	0.043
HERC2P5	ENSG00000260644.6	-3.547	0.014
OR4F17	ENSG00000176695.8	-3.426	0.029
COG5	ENSG00000284369.1	-3.371	5.0e-6
PPP1R11	ENSG00000206501.8	-3.306	3.6e-5
IGLV2-14	ENSG00000211666.2	-3.012	0.026
AC243948.1	ENSG00000276886.4	-2.786	0.004
MYT1L	ENSG00000186487.18	-2.472	0.022
DNAH3	ENSG00000158486.13	-2.434	0.014
CYP4F35P	ENSG00000265787.2	-2.018	0.043
CHI3L1	ENSG00000133048.12	-1.858	0.02
LRRN3	ENSG00000173114.12	-1.817	9.9e-4
MMP9	ENSG00000100985.7	-1.786	0.034
ZNF75A	ENSG00000162086.14	-1.691	5.0e-6
ARMC9	ENSG00000135931.17	-1.522	0.05
**Upregulated DEGs**
HLA-A	ENSG00000235657.9	9.333	3.6e-43
PSMB9	ENSG00000240508.7	5.751	3.6e-7
HLA-C	ENSG00000233841.11	5.572	4.3e-24
RPS9	ENSG00000274646.4	5.303	0.03
HBB	ENSG00000244734.4	5.201	4.8e-11
RPL10P9	ENSG00000233913.7	4.777	0.001
CLIC1	ENSG00000230685.6	4.162	0.001
HLA-DQB1	ENSG00000206302.10	3.77	1.5e-4
TRBV24-1	ENSG00000282730.1	3.701	0.015
OLR1	ENSG00000173391.8	3.489	6.5e-22
HBA2	ENSG00000188536.13	3.072	0.011
TRIM26	ENSG00000230230.7	2.772	0.005
MICB	ENSG00000234218.9	2.613	0.009
VSIG4	ENSG00000155659.14	2.432	0.003
NSFP1	ENSG00000282733.1	2.37	0.05
MMP2	ENSG00000087245.12	2.271	5.6e-4
CCL18	ENSG00000278167.2	2.024	0.013
ATXN7	ENSG00000285258.1	1.85	0.007
NAIP	ENSG00000276068.4	1.619	0.019
PTCH2	ENSG00000117425.13	1.502	0.025
KCTD12	ENSG00000178695.5	1.421	6.5e-4
SPOPL	ENSG00000144228.8	1.395	0.008
RAD17	ENSG00000152942.18	1.359	0.007
MS4A7	ENSG00000166927.12	1.297	0.03
TMEM167A	ENSG00000174695.9	1.164	0.014

**Figure 1.**
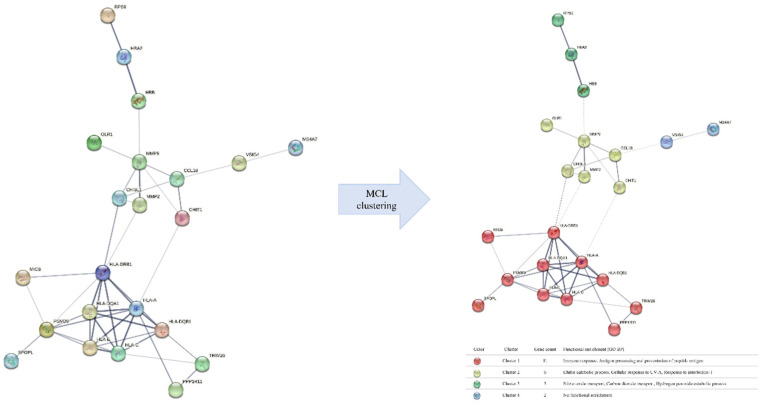
PPI network of identified DEGs, before and after MCL clustering algorithm.

The network produced by the STRING tool comprised 22 out of the 49 DEGs, with 44 edges between them, while the MCL clustering algorithm revealed 4 distinct protein clusters related to immune responses (HLA-A, HLA-C, HLA-DQA1, HLA-DQB1, HLA-DRB1, HLA-E, MICB, PPP1R11, PSMB9, SPOPL, TRIM26), the cellular response to UVA and interleykin-1 and chitin catabolic process (CCL18, CHI3L1, CHIT1, MMP2, MMP9, OLR1), the hydrogen peroxide catabolic process, and nitric oxide (NO) and carbon dioxide transportation (HBA2, HBB, RPS9), as highlighted in Figure 1. This evidence is previously supported [11]. It has been stressed that the accumulation of chitin in the body may play a significant role in AD, potentially triggering cognitive decline due to its neurotoxic properties and subsequent induction of neuroinflammation [24],[25]. The catabolic process of hydrogen peroxide, along with the transportation pathways of nitric oxide and carbon dioxide, are processes associated with oxidative stress. NO is closely associated with both protective and neurotoxic effects on the brain [26]. The consequences of oxidative stress have been observed in the brains of many AD patients [27]; however, the presence of such markers in their blood is not consistent among various studies [28].

Upon our analysis, the cluster of genes including HLA-A, HLA-DRB1, PSMB9, MMP9, CCL18, CHI3L1, HLA-C, HLA-DQA1, HLA-DQB1, HLA-E, CHIT1, HBB, HBA2, and VSIG4 exhibited the highest degrees of centrality and betweenness scores within the constructed network. More precisely, PSMB9 encodes for a proteasome subunit and participates in the processing of MHC class I peptides, thus contributing to the immunoproteasome function. Furthermore, PSMB9 has been associated with the regulation of cellular proteostasis under mitochondrial stress, thereby assuming a significant role in neurodegenerative diseases [29]. MMP9, a protein implicated in proteolysis, exhibited elevated expression levels in both the plasma and postmortem brain tissues of AD patients compared to healthy controls, suggesting an association with the disease's pathology [30],[31]. Numerous members within the matrix metalloproteinases (MMP) family have demonstrated involvement in AD, rendering them as promising biomarkers and potential targets for therapeutic intervention [32]. Chitinases, specifically CHI3L1 and CHIT1, have been reported as being dysregulated in patients, and their proposition as biomarkers for early neuroinflammation and the diagnosis of AD has been advanced [33].

**Figure 2.**
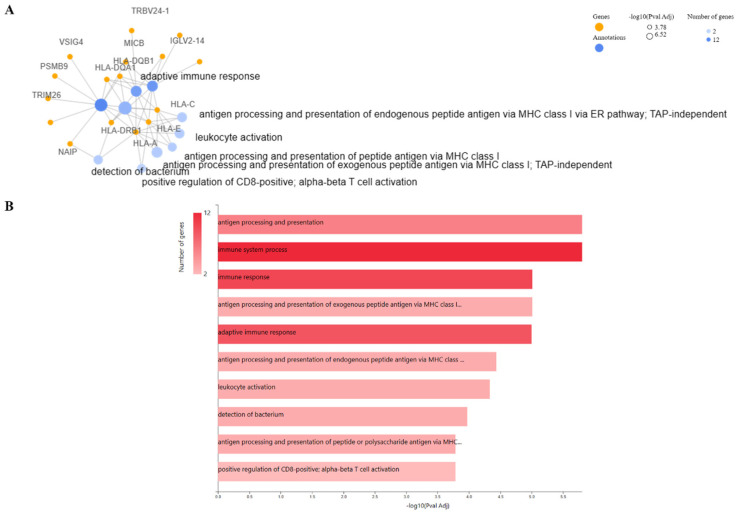
Pathway enrichment analysis. A) Gene-annotation cluster functional network and B) resulting pathways generated by GeneCodis platform.

**Figure 3.**
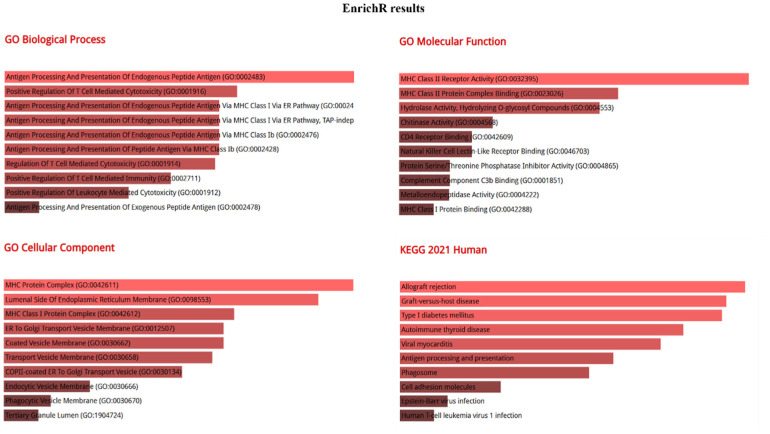
GO term and pathway enrichment analysis performed using Enrichr on DEGs including the top 10 enriched biological processes, molecular functions, cellular components and KEGG pathways for DEGs.

**Figure 4.**
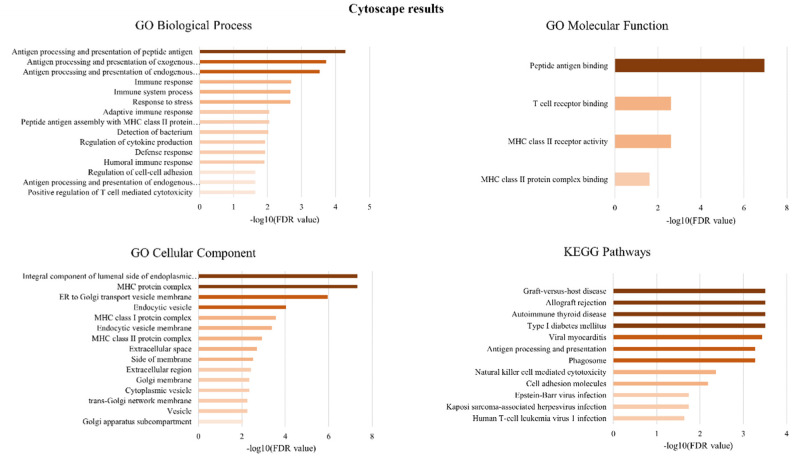
Pathway enrichment analysis. Gene Ontologies (biological processes, molecular functions, cellular components) and KEGG pathways related to DEGs, based on functional enrichment analysis provided by Cytoscape.

To identify perturbed pathways associated with DEGs, several platforms were utilized, and the ensuing results were subsequently cross-referenced for comparison. Figure 2 highlights the gene-annotation cluster functional network of DEGs and their relevant pathways (B) through GeneCodis implementation. Immune related pathways were the most highlighted (adaptive immune response, antigen processing and presentation of endogenous peptide antigen via MHC class I, leukocyte activation, detection of bacterium, positive regulation of CD-8 positive, and alpha-beta T cell activation). Immunity seems to have a critical effect on the brain. In recent years, growing evidence has revealed the dual roles of innate immune genes and cells in AD, thereby encompassing both advantageous and detrimental effects. Notably, peripheral immune cells such as T cells, with the ability to enter the brain, are implicated in the etiology and neuropathogenesis of AD [34]. In addition to peripheral T cell activation, patients diagnosed with AD display a notably higher percentage of activated CD8+ T cells in their cerebrospinal fluid (CSF) compared to their healthy older counterparts. This deviation is correlated with cognitive and neuroanatomical outcomes associated with AD [35]. To achieve a more robust outcome, we utilized the Enrichr and Cytoscape platforms, as depicted in Figure 3 and Figure 4, respectively, to explore the high-level functions and utilities of the biological system according to the exported genes. Endogenous and exogenous antigen binding has been revealed as the most significant relevant pathways from the platforms, including GeneCodis, based on the p- and FDR-values. Given that the analyses underscored genes associated with the presentation and processing of MHC molecules, these findings are expected and align with established knowledge. The enrichment analysis included GO terms divided into biological process (BP), molecular function (MF), and cellular component (CC) ontologies, as well as KEGG pathways. Our analysis showed that DEGs were mainly enriched in BP, including multiple antigen processing and the presentation of endogenous and exogenous peptide antigen via MHC class I and positive regulation of T cell mediated cytotoxicity processes. Cytoscape additionally showcased subsequent biological processes such as the immune system (adaptive and humoral) response and process, response to stress, peptide antigen assembly with MHC class II protein complex, detection of bacterium, regulation of cytokine production, and cell-cell adhesion.

Cellular component (CC) analysis from both platforms indicated that DEGs were significantly enriched in the MHC class I protein complex, the integral component of the lumenal side of endoplasmic reticulum membrane, and the ER to Golgi transport endocytic vesicle membrane. Moreover, phagocytic vesicle membranes and the tertiary granule lumen were exported by Enrichr, while the MHC class II protein complex, extracellular space, and the Golgi apparatus subcompartments were indicated via Cytoscape. Lastly, regarding the MF analysis, the outcomes observed from the two approaches were quite different. Consensus between both platforms affirmed the enrichment of DEGs in the MHC class II protein complex binding and the MHC class II receptor activity; however, Cytoscape revealed only two more enriched terms, namely T cell receptor binding and peptide antigen binding, while Enrichr revealed the following ontologies among them: hydrolase activity, hydrolyxing O-glycosyl compounds, chitinase activity, CD4 receptor activity, natural killer cell lectin-like receptor binding, protein Ser/Thr phosphatase inhibitor activity, complement component C3b binding, metalloendopeptidase activity, and MHC class I protein binding (Figure 3). Regarding KEGG pathway analyses, both platforms concluded that DEGs were mainly enriched in pathways such as autoimmune thyroid disease, antigen processing and presentation, phagosome, cell adhesion molecules, and natural killer cell mediated cytotoxicity, ontologies were also indicated using Cytoscape (Figure 4).

The development of AD involves unclear mechanisms, with recognized risk factors including age, genetics, metal exposure, traumatic brain injury, lifestyle, malnutrition, diabetes, immune dysfunction, vascular disease, and psychiatric factors. Causative factors may include chronic stress, oxidative stress, and inflammation. Recent evidence supports the roles of chronic stress, glucocorticoids, oxidative stress, and inflammation in both initiating and progressing AD [36]. Oxidative stress indicators in AD individuals include elevated levels of oxidized proteins, advanced glycation end products, lipid peroxidation end products, and the formation of toxic entities. Age-related memory impairments in AD are associated with a decline in antioxidant defense mechanisms, notably the compound glutathione (GSH), which are responsible for maintaining cellular redox potential [37]. AD involves intricate molecular mechanisms. Key processes include the accumulation of amyloid plaques, primarily composed of amyloid-beta (Aß) derived from the amyloid precursor protein (APP), around blood vessels and neurons. The principal AD hallmarks include Aβ and its plaques in the brain's cortical regions. Additionally, microtubule-associated Tau proteins, initially structural proteins, undergo hyperphosphorylation, thus transforming into toxic aggregated proteins known as neurofibrillary tangles, contributing to AD pathophysiology [38]. The Tau hypothesis also suggests that in AD, Tau tangles precede Aβ plaques, and Tau phosphorylation and aggregation are the main triggers of neurodegeneration. The amyloid hypothesis suggests that amyloid-β accumulation initiates Alzheimer's progression. While initially viewed as a neurodegenerative disorder, neuroinflammation is now recognized alongside amyloid-β and tau pathology. Genetic and functional evidence underscores the active role of the brain's innate immunity in Alzheimer's pathogenesis.

In our analysis, the identified gene cluster exhibited important factors such as PSMB9, which encodes a proteasome subunit and is integral to immunoproteasome function, associated with neurodegenerative disorders. Furthermore, the chitinases CHI3L1 and CHIT1 are being explored as prospective biomarkers for early-stage neuroinflammation and for the diagnosis of AD. DEGs were mainly associated with antigen processing, MHC class I presentation, T cell-mediated cytotoxicity, cytokine regulation processes, and the immune and stress responses were predominantly enriched in pathways including autoimmune thyroid diseases, cell adhesion molecules, and antigen processing and presentation. The dysfunction of the immune system is closely associated with AD progression. Immune cells, particularly the microglia in the central nervous system, exhibit dynamic changes and diverse functions throughout different disease stages. Peripheral immune cells can be recruited into the CNS, thus contributing to AD development [39]. Early clinical studies on immune dysregulation focused on peripheral inflammatory markers, indicating that changes in these markers precede cognitive and symptomatic outcomes in late life. Fundamental epidemiological research highlighted the importance of baseline levels of C-reactive protein, IL-6, and the production of TNF and IL-1β by peripheral blood mononuclear cells in predicting pathological outcomes [40]. Peripheral inflammatory markers in older adults without symptoms have shown connections with various clinical factors and biomarkers, including cognition, brain structure, functional brain connectivity, and future amyloidosis. However, clarifying the temporal progression and clinical significance of peripheral immune dysregulation in the context of symptomatic AD has posed a considerable challenge [41]. Dementia patients show impaired cortisol regulation, suggesting a dysfunctional HPA axis. Elevated cortisol levels correlate with a faster disease progression, indicating an overactive HPA axis in advanced stages, likely accelerating the condition [42]. Chronic stress prompts the immune system to release pro-inflammatory cytokines, thus influencing neural activity in the brain. This can lead to a dysregulated immune response, thus contributing to inflammation and impacting various physiological processes, including neural function [43].

The hippocampus, crucial for memory, is central to AD, with atrophy as a key biomarker. It houses cortisol receptors, namely high-affinity mineralocorticoid (MR) receptors, that provide protection and promote resilience, while low-affinity glucocorticoid (GR) receptors may contribute to neuronal death. Maintaining a balance between these receptors is vital for proper hippocampal function [44]. Previous machine learning analyses across the cortex and hippocampus regions of AD samples showed an involvement in glutamatergic synapses, dendrite and cell surfaces, and cerebral cortex GABAergic interneuron migration [45]. Continual midlife stress can upset the delicate balance, thus leading to lasting hippocampal dysfunction. Those vulnerable to psychological distress face a higher risk of MCI [46]. Stress disrupts the delicate balance, reducing hippocampal thickness in rats exposed to physical and psychological stressors, atrophy initially hit CA1 (physical stress), followed by CA3 and the dentate gyrus. Although brain effects normalized after physical stress cessation, dentate gyrus atrophy persisted even after psychological stress ended [47]. While human studies have predominantly concentrated on plasma cytokine levels when investigating immune dysfunction and AD stages, the evaluation of monocyte phenotypes and functions have concurrently uncovered a contributory role in the development of AD dementia [48].

Comparing our results to the existing literature, we find both confirmatory and novel findings. Consistent with previous studies, our analysis supports the involvement of immune-related pathways and genes in AD pathogenesis. Specifically, it has been reported by many studies that the levels of different immune cells differ between patients and healthy controls [49],[50]. DEGs in those studies seem to be involved in cytokine receptor activity and neutrophil activation, which are important pathways for the stimulation of the immune system [50]. Similarly, the up-regulation of MMP9 and chitinases suggests their involvement in neuroinflammation and neurodegeneration, thus corroborating previous findings [30],[51]. However, our study provides novel insights into the specific genes and pathways implicated in immune dysregulation and oxidative stress in AD. For instance, the role of immunoproteasome function and its association with mitochondrial stress have been previously associated with AD [52],[53]; specifically, the PSMB9 gene has not been reported again in humans, thus highlighting its potential as a therapeutic target for AD.

It is obvious, that our findings validate and enrich the results of previous studies, thus contributing to the better understanding of the immunity's involvement in AD. However, there are several limitations to our study concerning the fact that the analysis was based on a single dataset with a limited number of subjects and only data derived from one tissue. Additionally, there are other factors that cannot be accounted for, such as the sample characteristics, such as differences in age, sex, race, ethnicity, education, comorbidities, or concurrent medications, which can cause variations in gene expression. Because of this, careful consideration should be given to the interpretation of the results. Finally, more research on the topic is crucial, both utilizing bioinformatic methods in a bigger group of datasets and validating existing in silico results with wet lab experiments.

## 4. Conclusions

Within the field of neurodegenerative diseases, numerous studies have examined the molecular mechanisms through which systemic inflammation may lead to long-term cognitive decline and contribute to the process of neurodegeneration. AD displays phases without symptoms, and most immunological data rely on cross-sectional analyses, pitting healthy community members against conveniently sampled symptomatic adults from specialized clinics. Nonetheless, these cases may not precisely depict the intricate range of AD presentations. Overall, the present study emphasizes the critical effect of the immune peripheral system responses in AD. Peripheral blood-derived innate immune cells and neuroinflammation; moreover, stress-related factors, both within the brain and throughout the body, can significantly influence the development and progression of the disorder. Understanding the complex relationship between immunity and AD is crucial to develop efficient interventions and treatments. Hence, a pivotal progression in the field entails the capability to comprehensively evaluate longitudinal immunological and AD data within cohorts characterized by phenotypic diversity and demographic heterogeneity.
